# The Prevalence of Self-Reported Tuberculosis in the Andaman and Nicobar Islands, India: Evidence from the NFHS-IV and V

**DOI:** 10.3390/tropicalmed8100464

**Published:** 2023-10-03

**Authors:** Kannan Thiruvengadam, Rajendran Krishnan, Malaisamy Muniyandi

**Affiliations:** ICMR-National Institute for Research in Tuberculosis, Chennai 60031, India; kannan.t@icmr.gov.in (K.T.);

**Keywords:** tuberculosis, infectious diseases, point prevalence of tuberculosis, self-reported tuberculosis, National Family Health Surveys

## Abstract

Introduction: Since 1992, many rounds of the National Family Health Surveys have produced a significant quantity of data in India. The magnitude of the tuberculosis (TB) burden in Andaman and Nicobar Island can be better understood with this data. The household-level information on self-reported TB may provide useful information on the prevalence and distribution of TB as well as care-seeking behaviour. The primary objective is to analyse the data from the NFHS-IV and NFHS-V to understand the prevalence of self-reported TB as well as healthcare-seeking patterns for TB in the Andaman and Nicobar Islands. Methodology: We performed secondary data analysis of NFHS-IV and NFHS-V data. After taking into consideration the survey’s cluster design and sampling weights, the prevalence was estimated. The association of identified factors with self-reported TB was investigated using the chi-square and logistic regression models. Results: The point prevalence of self-reported TB was 615 (418, 873) and 221 (122, 367) in the NFHS-IV and NFHS-V, respectively (*p* = 0.012). The elderly, those from rural areas, those belonging to a tribe, and those with a poor wealth index were more likely to report TB. Self-reported TB prevalence was higher in the Nicobar district. There is an increase in a significant proportion of individuals not seeking care. Conclusion: The NFHS-IV and NFHS-V show a decline in self-reported TB, which is consistent with national estimates. However, the enhanced TB case detection in individuals at high risk of TB among the Nicobar districts and tribal communities could significantly contribute to the fight against tuberculosis. Improved awareness of TB could improve care seeking for TB.

## 1. Introduction

Tuberculosis (TB) has existed with us for thousands of years and continues to be the world’s leading killer among infectious diseases [1]. Geographically, South-East Asia’s WHO regions had the majority of TB cases (45.0%) in 2021. Between 2005 and 2019, the annual number of deaths due to TB decreased globally, but in 2020 and 2021, this trend was reversed [1]. India accounts for around 27% of the global total with high heterogeneity in the distribution of TB burden within the country [2]. The Indian government, on the other hand, wants to eliminate tuberculosis (TB) by 2025 [3]. To achieve TB eradication, there should only be ten instances of the disease per 100,000 population [4].

The Andaman and Nicobar Islands are an archipelago of over 500 islands, some of which have populations in very remote and inaccessible islands. Very little information is available about the problem of tuberculosis, and a few studies have shown the existence of inadequate healthcare services despite high demand [5]. Noticeably, this population was not surveyed in the national TB prevalence survey conducted between 2019 and 2021 [6], although the islands’ population has been mostly stable and the programme has been successfully implemented. Tuberculosis accounts for 29% of the deaths due to infectious aetiology in the Andaman and Nicobar Islands. With an increased effort on active case finding, the rate of notification has tended to decrease. However, the presumptive examination rate and the number of cases that need to be tested to find TB positives have increased [7]. Most research reviews indicate that the major factor contributing to the transmission of TB disease is the limited accessibility and availability of TB services [8]. It is observed that in low- and middle-income countries, vulnerable populations, such as miners, prisoners, the elderly, those with HIV, or those residing in isolated areas, are often not diagnosed or treated for TB [9].

A major limitation of the current estimates given by the TB programme is incomplete notifications, which could underestimate the TB burden [10] Additionally, there is no precise information on care-seeking behaviour for TB. Most importantly, these islands are home to six primitive tribes, which constitute about 10% of the population and immigrants from the mainland of India. There is evidence that TB is more prevalent, particularly among the tribes [11]. Compared to the national average (312 per 100,000), the prevalence of TB is greater among tribal people (703 per 100,000) [12]. Tribal groups account for around 10.4% of all TB patients who have been identified in India [11].

To understand the magnitude of TB in Andaman and Nicobar Island, the National Family Health Survey Fourth Round (NFHS-IV) [13] and Fifth Round (NFHS-V) [14] data were used. The household-level information on self-reported TB (cases on treatment who confirmed their treatment status during the survey) may provide helpful information on the prevalence and distribution of TB as well as the care-seeking pattern among those with TB [15].

## 2. Materials and Methods

### 2.1. Source of Data

The data pertaining to the Andaman and Nicobar Islands from the NFHS-IV and NFHS-V conducted in 2015–2016 and 2019–2021 were analysed. The Ministry of Health and Family Welfare, Government of India, conducts NFHS surveys, which are extensive surveys with robust sample techniques. This NFHS is India’s version of the Demographic and Health Survey (DHS). The Details of the sampling method and survey questionnaires are available from the NFHS-IV [13] and NFHS-V [14] reports. These two surveys were made to produce precise district-level estimates as well as estimates for subgroups of districts, such as the disaggregation of urban and rural areas.

### 2.2. Data Collection

In the Andaman and Nicobar Islands, 10,288 and 9388 people from 2413 and 2623 households were questioned utilising a structured interview schedule and computer-assisted personal interview. The TB information came from the household schedule, which contains vital information about each member of the household [13,14]. The screening question “Does any regular household member have tuberculosis?” was followed by questions on whether the suffering individual had medical treatment for TB and whether it came from the public, private, or a combination of both sectors [13,14]. We also used information retrieved from the NIKSHAY portal [16], which the Indian government launched on 4 June 2012.

### 2.3. Data Analysis

We described the characteristics of study participants in unweighted frequencies and percentages accounted for sampling weights. The crude prevalence and the 95% confidence interval were calculated with an exact binomial formula, and the adjusted prevalence was estimated using logistic regression, accounting for the cluster design and sampling weights of the survey [17]. The chi-square test was performed after adjusting for a survey design to observe the association of the self-reported TB against background factors like age, gender, place of residence, level of education, tribal population, and union territory level of wealth index classification. We estimated the odds ratio and adjusted it for the odds of reporting having TB using a logistic regression model with the background variables as explanatory variables. The association of background factors with the choice of health facilities was also assessed. A *p*-value of <0.05 was considered statistically significant. All analyses were performed in STATA/MP version 16.0 (STATA Corporation LLC, College Station, TX, USA).

## 3. Results

### 3.1. Study Population

**Figure 1:**
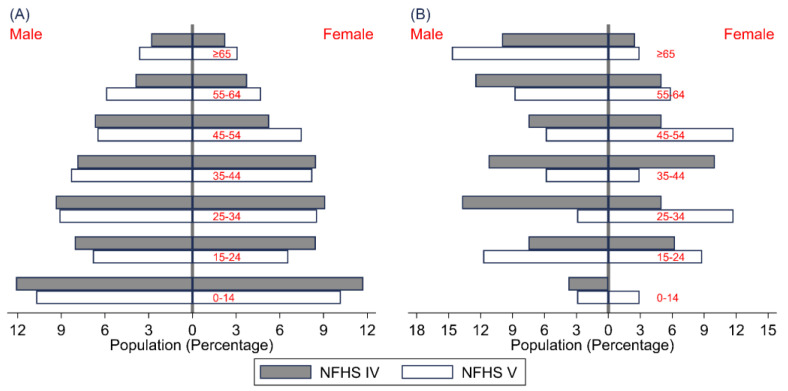
Age Pyramid of Participants (**A**) and Self-reported Tuberculosis Participants (**B**) of NFHS-IV and NFHS-V.

Out of the 10,288 and 9388 individuals interviewed in the NFHS-IV and NFHS-V, 80 (0.6%) in NFHS-IV and 148 (0.4%) in NFHS-V reported that a household member suffered from TB. The observed proportion of self-reported TB significantly increased from NFHS-IV to NFHS-V, (*p* = 0.012). The participant age pyramid (Figure 1A) demonstrates that a similar representation was seen in both surveys. The percentage of self-reported TB seen throughout the age groups, however, has significantly changed between surveys.

### 3.2. Prevalence of TB

The data show that the crude point prevalence was 778 (617, 967) and 362 (251, 506) per 100,000 population. After adjusting for the survey design and sampling weights, the prevalence of self-reported TB was 615 (418, 873) and 221 (122, 367) in the observed NFHS-IV and NFHS-V (Table 1). These numbers indicate a significant decline in self-reported TB of about 64.1% from NFHS-IV to NFHS-V. There is also a considerable decline shown in all of the subgroups, except for the age of 45 to 54 (Table 1). Between the ages of 15 to 34 and 45 to 54, the proportion of women with TB has increased. Elderly males also have shown an increase in TB (Figure 1B).

### 3.3. Factors Associated with Self-Reported TB

**Figure 2:**
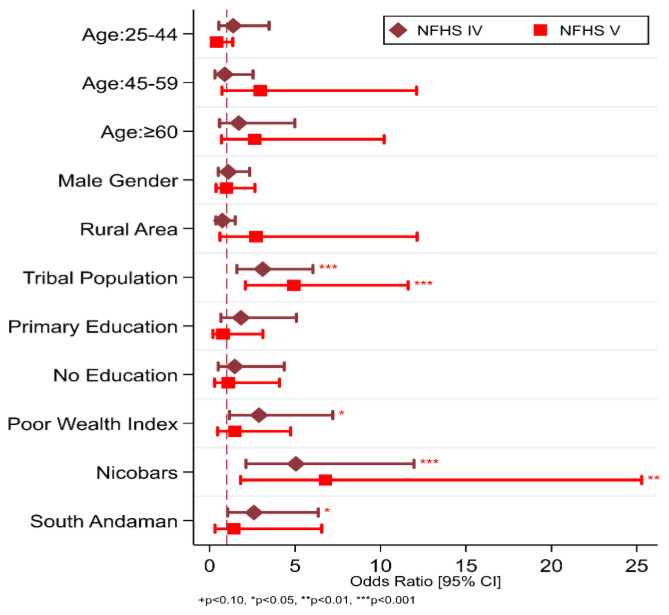
Factor associated with Self-Reported TB in Andaman and Nicobars Island in the NFHS-IV and NFHS-V.

We also assessed the factors that are associated with the self-reported prevalence of TB. The findings show that the odds of having TB are considerably higher in the tribes in NFHS-IV and NFHS-V (OR: 3.10, 95% CI: 1.59–6.05 and 4.92, 95% CI: 2.09–11.62, respectively). Being in the poorer/poorest wealth index was associated with greater odds of developing TB in NFHS-IV (OR: 2.89, 95% CI: 1.16–7.21) but was not shown to be significant in NFHS-V. Similar to the wealth index, the South Andaman district was only significant in NFHS-IV (OR: 2.89, 95% CI: 1.16–7.21) (Figure 2). In both NFHS-IV (prevalence per 100,000 population 1322 (861, 1940)) and NFHS-V (prevalence per 100,000 population 852 (535, 1286), the Nicobar district was consistently shown to have higher self-reported TB prevalence (Table 1). Compared to other districts, those in Nicobar district had higher odds (OR: 5.05, 95% CI: 2.13–11.96 and 6.78, 95% CI: 1.82–25.28, respectively) of having TB in both surveys (Figure 2).

Table 2 displays the overall treatment-seeking behaviour of the population of the Andaman and Nicobar Islands, demonstrating intriguing patterns across the factors. The behaviour remained consistent in NFHS-IV and NFHS-V. It was found that health facility preferences were not influenced by the age, gender, or the tribal status of the participants. Participants who were from urban areas (*p* < 0.001), had higher levels of education (*p* < 0.001), and had higher wealth indices (*p* < 0.001) were more likely to use private facilities. The South Andaman District participants preferred private facilities, which may have been related to availability (*p* < 0.001).

**Figure 3:**
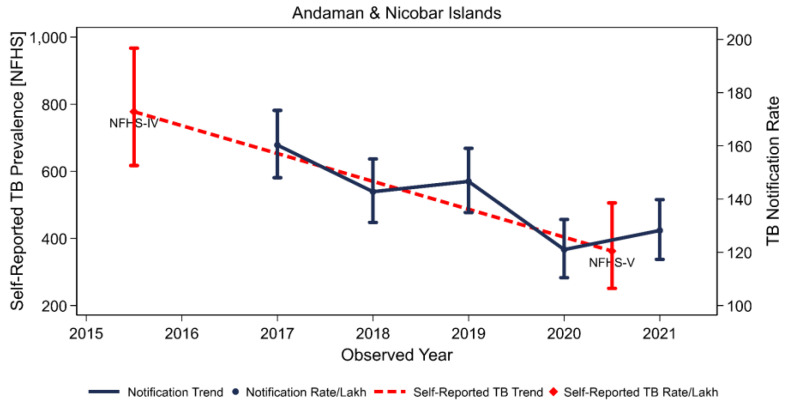
The TB notification trend and the prevalence of self-reported TB in Andaman and Nicobar Island.

In both surveys, 85.0% and 78.7% of self-reported TB patients received care from the public sector, while the remaining patients received care from the private sector or both the public and private sectors. There was a significant dependence on public resources for TB treatment in the Nicobars and North and Middle Andaman districts, while some patients were being treated in the private sector in the south. There was a higher proportion of TB participants in NFHS-V (11.1%) than in NFHS-IV (7.2%) who were reported to not be receiving treatment; however, it was not significant. The self-reported TB prevalence rate by the NFHS exhibited a similar trend to the TB notification rate, as shown in Figure 3. Except for the Nicobar districts and their tribal populations, all of these results demonstrate that there has been a decrease in TB cases in the Andaman and Nicobar Islands.

## 4. Discussion

We observed almost similar patterns of self-reported TB and their associated factors through this comprehensive assessment. The current estimate of TB prevalence is not a precise representation of the burden of TB even though it was from a large-scale survey with a good representative sampling procedure. However, there was a significant decrease in the proportion of self-reported TB during 2019–2021. The national TB prevalence recorded in the most recent national-level survey was almost equal to the crude prevalence in the NFHS-V [6]. It was also lower than the national estimate published in the 2022 Global TB Report but was close to the estimate in [1].

The pattern of prevalence was similar in both surveys, and it rose as people’s ages increased. This is in line with research that looked at the incidence and death rates of TB during the previous three decades, where the study reported that overall, the incidence of TB increased with the age group [18]. Factors such as the reactivation of latent TB infection, a weakened immune system, coexisting health conditions, prolonged exposure to TB, and a delayed diagnosis may contribute to an increase in TB prevalence as age increases. A detailed and focused study is needed to better understand these causal factors. A systematic review of the prevalence of pulmonary tuberculosis in India reported that males had a higher pooled prevalence of bacteriologically positive pulmonary tuberculosis than females [19,20]. We found that the males had a higher prevalence in NFHS-IV and were slightly equal to females in the NFHS-V. According to the literature and the current data, rural regions have a much greater frequency of TB than metropolitan areas [19,20,21,22]. With an alarming 703 [23] and 432 [12] cases per 100,000 individuals, or roughly twice as many as the Indian average [6], TB has been recognised as a serious public health concern across all Indian tribes. A high level of care is needed for the 75 various extremely marginalised tribal populations who have a higher TB incidence, since doing so will significantly lessen India’s TB burden [12,23,24,25,26]. People without education had the greatest frequency of TB, while the prevalence of TB varied with educational level [20,27]. The knowledge, awareness, health literacy, healthcare knowledge, lifestyle, behaviour, and socioeconomic status were linked to education level. Hence, the education levels had an impact on the disease prevalence. However, the lack of a trend in this group, regardless of education level, points to a lack of TB awareness in this community. As expected, those in poverty had a higher risk of contracting TB than those in the top quintile of wealth class [27,28]. Despite a strong commitment and strategy for TB elimination by 2025 [29], there exists a gap between detection and treatment. This gap can be attributed to several factors, such as a scarcity of health services and facilities in this resource-constrained area [30].

The findings suggest the importance of determining the TB burden in the Andaman and Nicobar Islands, especially among the tribal community and the Nicobar district. To reach the unreached and obtain an accurate assessment of the disease burden, methodological approaches must be taken into account. To improve Andaman and Nicobar Islanders’ access to healthcare facilities, it is also crucial to comprehend the health-seeking behaviours of this population, particularly regarding TB symptoms. To strengthen the model for India, it is important to investigate the possible involvement of this community in TB control initiatives, including the identification and referral of symptomatic patients for care. The public and private health sectors in India must be successfully engaged to expedite the drop in the TB burden [31].

## 5. Limitations

This manuscript presents a comprehensive assessment of TB prevalence in the Andaman and Nicobar Islands. It is based on data from the fourth and fifth rounds of the National Family Health Survey using a weighted analysis. We identified predictors of self-reported TB from a large and representative dataset. However, one of the major limitations of this study is that it only considers predetermined variables. In contrast, there are numerous other factors beyond the scope of the NFHS that influence TB. It is also important to note that self-reporting can lead to an underestimation of TB prevalence.

## 6. Conclusions

The study findings from both surveys indicate that there is a decrease in the proportion of self-reported TB, and the prevalence aligns with the national averages. There are recognised risk factors, such as the site of residence, educational attainment, being a tribe member from a remote area, and poor wealth index, that are substantially linked with self-reported tuberculosis as well. To reduce the burden of tuberculosis among this population, a focused strategy is needed, including addressing the gaps in access to healthcare, community-based interventions, targeted screening programs, nutritional supports, health education programs, and good surveillance, since these are the only ways to better understand the TB burden in tribal and resource-limited environments such as the Nicobar group of islands. Targeted interventions in the tribal communities of the Andaman and Nicobar Islands, as well as in the Nicobar districts, may significantly reduce TB incidence.

## Figures and Tables

**Table 1:** Self-Reported Tuberculosis Prevalence in Andaman and Nicobars Island based on the National Family Health Survey IV and V.

	NFHS-IV						NFHS-V					
	Self-Reported TB				Crude Prevalence (95% CI)	Adjusted TB Prevalence (95% CI)	Self-Reported TB				Crude Prevalence (95% CI)	Adjusted TB Prevalence (95% CI)
	Popn-10,288		TB-80				Popn-9388		TB-34			
	N	%	n	%			N	%	n	%		
Age Group (in Years)												
0 to 14	2451	23.9%	3	9.0%	122 (25, 357)	234 (19, 967)	1963	20.9%	2	5.9%	102 (12, 368)	62 (5, 260)
15 to 24	1684	16.7%	11	25.4%	653 (327, 1166)	946 (329, 2116)	1260	13.4%	7	20.6%	556 (224, 1141)	315 (76, 855)
25 to 34	1900	19.8%	15	23.3%	789 (443, 1299)	723 (310, 1428)	1660	17.7%	5	14.7%	301 (98, 702)	79 (26, 182)
35 to 44	1692	16.4%	17	19.2%	1005 (586, 1604)	718 (250, 1606)	1555	16.6%	3	8.8%	193 (40, 563)	53 (11, 154)
45 to 54	1243	11.7%	10	6.6%	805 (386, 1475)	345 (100, 851)	1316	14.0%	6	17.6%	456 (167, 990)	581 (140, 1570)
55 to 64	794	7.1%	14	7.5%	1763 (967, 2941)	643 (199, 1535)	998	10.6%	5	14.7%	501 (163, 1165)	219 (54, 588)
≥65	524	4.5%	10	9.0%	1908 (919, 3482)	1230 (359, 3017)	636	6.8%	6	17.6%	943 (347, 2042)	616 (142, 1706)
Gender												
Male	5253	50.5%	53	52.9%	1009 (757, 1318)	642 (370, 1034)	4800	51.1%	18	52.9%	375 (222, 592)	219 (101, 414)
Female	5035	49.5%	27	47.1%	536 (354, 779)	588 (310, 1011)	4588	48.9%	16	47.1%	349 (199, 566)	223 (85, 473)
Residence Area												
Urban	1692	42.0%	11	49.9%	650 (325, 1160)	724 (362, 1292)	1952	20.8%	2	5.9%	102 (12, 370)	107 (13, 386)
Rural	8596	58.1%	69	50.2%	803 (625, 1015)	535 (349, 785)	7436	79.2%	32	94.1%	430 (295, 607)	289 (153, 496)
Tribal Population												
Non-Tribes	7156	93.2%	32	81.6%	447 (306, 631)	539 (338, 815)	6822	72.7%	9	26.5%	132 (60, 250)	164 (67, 334)
Tribes	3132	6.8%	48	18.4%	1533 (1132, 2027)	1653 (932, 2703)	2566	27.3%	25	73.5%	974 (631, 1435)	801 (493, 1229)
Education												
No Education	3502	31.9%	25	30.3%	714 (463, 1052)	587 (273, 1098)	3087	32.9%	15	44.1%	486 (272, 800)	260 (103, 543)
Primary	4953	47.0%	44	55.9%	888 (646, 1191)	731 (412, 1198)	4419	47.1%	12	35.3%	272 (140, 474)	185 (60, 433)
Secondary or Higher	1833	21.2%	11	13.8%	600 (300, 1071)	400 (132, 923)	1882	20.0%	7	20.6%	372 (150, 765)	237 (56, 648)
Wealth Index												
Poorest/Poorer	5441	40.1%	53	57.9%	974 (730, 1272)	889 (513, 1432)	5034	53.6%	24	70.6%	477 (306, 709)	241 (131, 407)
Middle	2236	19.9%	17	23.3%	760 (444, 1215)	718 (315, 1395)	1749	18.6%	5	14.7%	286 (93, 666)	278 (43, 914)
Richer/Richest	2611	40.0%	10	18.8%	383 (184, 703)	289 (104, 638)	2605	27.7%	5	14.7%	192 (62, 447)	172 (42, 462)
Districts												
Nicobars	3952	7.0%	52	15.1%	1316 (984, 1722)	1322 (861, 1940)	3223	34.3%	25	73.5%	776 (503, 1143)	852 (535, 1286)
North and Middle	3073	26.8%	8	11.4%	260 (112, 512)	265 (104, 552)	3014	32.1%	4	11.8%	133 (36, 339)	127 (22, 400)
South	3263	66.2%	20	73.5%	613 (375, 945)	681 (404, 1074)	3151	33.6%	5	14.7%	159 (52, 370)	180 (57, 425)
Overall												
	10,288	100.0%	80	0.6%	778 (617, 967)	615 (418, 873)	9388	100.0%	34	0.4%	362 (251, 506)	221 (122, 367)

TB was defined as the individual listed as suffering from TB. Prevalence was reported per 100,000 population. Crude prevalence and their confidence intervals were calculated with exact binomial probability theory. Adjusted prevalence and their confidence intervals were calculated using the log-binomial link function after adjusting for clustering and sampling weights. The ‘n’ denotes an unweighted number of observations, and the percentage accounts for sampling weights.

**Table 2:** General Treatment-seeking patterns of the Andaman and Nicobars Island People regarding Where household members generally go for treatment.

Factors	NFHS–IV					NFHS-V				
	Public		Private		*p*-Value	Public		Private		*p*-Value
	N	%	n	%		n	%	n	%	
Age Group (in Years)										
0 to 14	2458	24.0%	37	21.8%	0.838	1932	20.9%	31	14.1%	0.089
15 to 24	1708	16.8%	23	13.1%		1215	13.9%	45	20.5%	
25 to 34	1894	19.7%	42	21.9%		1625	18.9%	35	16.4%	
35 to 44	1687	16.4%	28	16.9%		1520	16.4%	35	15.8%	
45 to 54	1229	11.6%	22	13.0%		1277	13.5%	39	16.7%	
55 to 64	787	7.1%	15	8.0%		976	10.1%	22	10.4%	
≥65	523	4.4%	9	5.3%		622	6.3%	14	6.1%	
Gender										
Male	5239	50.6%	87	47.9%	0.279	4692	51.5%	108	48.5%	0.223
Female	5047	49.4%	89	52.1%		4475	48.5%	113	51.5%	
Residence Area										
Urban	1569	40.4%	134	83.1%	<0.001	1783	35.4%	169	77.7%	<0.001
Rural	8717	59.6%	42	16.9%		7384	64.6%	52	22.3%	
Tribal Population										
Non-Tribes	7127	93.1%	172	97.4%	0.300	6613	90.9%	209	95.1%	0.377
Tribes	3159	7.0%	4	2.6%		2554	9.2%	12	5.0%	
Education										
No Education	3511	32.1%	45	24.8%	0.024	3039	32.0%	48	21.9%	<0.001
Primary	4962	47.2%	69	42.1%		4355	46.5%	64	28.5%	
Secondary or Higher	1813	20.7%	62	33.0%		1773	21.5%	109	49.6%	
Wealth Index										
Poorest/Poorer	5506	41.2%	25	11.3%	0.007	5021	41.6%	13	6.1%	<0.001
Middle	2221	19.8%	38	21.6%		1712	20.2%	37	17.1%	
Richer/Richest	2559	39.0%	113	67.1%		2434	38.3%	171	76.9%	
Districts										
Nicobars	3994	7.2%	1	0.0%	<0.001	3223	8.8%	0	0.0%	<0.001
North and Middle Andaman	3148	27.8%	4	1.0%		3007	30.6%	7	1.8%	
South Andaman	3144	65.0%	171	98.9%		2937	60.6%	214	98.2%	
Self-reported TB Disease										
Non-TB	10,207	99.4%	175	99.4%	0.943	9134	99.8%	220	99.5%	0.432
TB	79	0.6%	1	0.7%		33	0.2%	1	0.5%	
TB Treatment Service Providers										
None/No treatment	2	7.5%	0	0.0%	0.978	3	12.1%	0	0.0%	0.003
Yes, public sector only	74	84.4%	1	100.0%		29	86.3%	0	0.0%	
Yes, private sector only	1	4.0%	0	0.0%		0	0.0%	1	100.0%	
Yes, both sectors	2	4.1%	0	0.0%		1	1.6%	0	0.0%	
Overall										
	10,286	96.5%	176	3.6%		9167	95.7%	221	4.3%	

The ‘n’ denotes the unweighted number of observations and the percentage accounting for sampling weights. The chi-square test was performed after adjusting for the survey design.

## Data Availability

All data generated during this study are included in this published article.
